# A Rice Receptor-like Protein Negatively Regulates Rice Resistance to Southern Rice Black-Streaked Dwarf Virus Infection

**DOI:** 10.3390/v15040973

**Published:** 2023-04-15

**Authors:** Fengmin Wang, Weiqi Song, Chaorui Huang, Zhongyan Wei, Yanjun Li, Jianping Chen, Hehong Zhang, Zongtao Sun

**Affiliations:** State Key Laboratory for Managing Biotic and Chemical Threats to the Quality and Safety of Agro-Products, Key Laboratory of Biotechnology in Plant Protection of MOA of China and Zhejiang Province, Institute of Plant Virology, Ningbo University, Ningbo 315211, China

**Keywords:** rice, receptor-like protein, pattern-triggered immunity, southern rice black-streaked dwarf virus, transcriptome

## Abstract

Plants rely on various receptor-like proteins and receptor-like kinases to recognize and defend against invading pathogens. However, research on the role of receptor-like proteins in plant antiviral defense, particularly in rice–virus interactions, is limited. In this study, we identified a receptor-like gene, *OsBAP1*, which was significantly induced upon infection with southern rice black-streaked dwarf virus (SRBSDV) infection. A viral inoculation assay showed that the OsBAP1 knockout mutant exhibited enhanced resistance to SRBSDV infection, indicating that OsBAP1 plays a negatively regulated role in rice resistance to viral infection. Transcriptome analysis revealed that the genes involved in plant–pathogen interactions, plant hormone signal transduction, oxidation–reduction reactions, and protein phosphorylation pathways were significantly enriched in *OsBAP1* mutant plants (*osbap1-cas*). Quantitative real-time PCR (RT-qPCR) analysis further demonstrated that some defense-related genes were significantly induced during SRBSDV infection in *osbap1-cas* mutants. Our findings provide new insights into the role of receptor-like proteins in plant immune signaling pathways, and demonstrate that OsBAP1 negatively regulates rice resistance to SRBSDV infection.

## 1. Introduction

Rice, as one of the world’s major food crops, is widely cultivated in more than 100 countries around the world [1,2]. Rice virus disease has caused a substantial reduction in crop yield and seriously endangered the safety of production [3]. Currently, more than ten species of rice viruses have been identified, all of which are mainly transmitted by the insect vectors, rice planthoppers, or leafhoppers [4,5]. Among these viruses, the southern rice black-streaked dwarf virus (SRBSDV) is a severe threat to rice food security in China. SRBSDV is a double-stranded RNA virus and belongs to the genus *Fijivirus*, family *Reoviridae* [3,6,7]. It has an icosahedral sphere with ten double-stranded RNAs (dsRNAs) encoding a total of 13 open reading frames (ORFs), including six structural proteins (P1, P2, P3, P4, P8, and P10) and seven non-structural proteins (P5-1, P5-2, P6, P7-1, P7-2, P9-1, and P9-2) [3,8]. SRBSDV is efficiently transmitted by the *Sogatella furcifera* (WBPH) in a persistent, circulative–propagative manner [9,10]. Infected plants exhibit symptoms, such as severe dwarfism, leaf stiffness, stunting, and an inability to pull nodes and spikes, which significantly affect rice yield [3,10]. Due to the high migratory ability of the WBPH, the virus exhibits characteristics of high explosiveness, rapid spread, difficult prevention and control, and significant damage and losses [5,11].

Plants have developed sophisticated defense mechanisms to protect themselves against a diverse range of pathogens, including bacteria, fungi, and viruses [12]. The plant innate immune system can be divided into two major categories: pathogen-associated molecular pattern (PAMP)-triggered immunity (PTI) and effector-triggered immunity (ETI) [13,14,15,16,17,18]. The first line of defense in plants is PTI, which recognizes PAMPs using pattern recognition receptors (PRRs) and initiates signaling cascades to activate the plant’s first immune response. This process, known as PTI, restricts the proliferation of most potential pathogens [19,20,21]. Plants possess hundreds of pattern recognition receptors located on the cell membrane that specifically recognize external pathogens and transmit signal molecules through the cell membrane to the intracellular compartment, allowing the plant to initiate a series of biochemical responses to against relevant molecular patterns [20]. The two main classes of pattern recognition receptors are receptor-like kinases (RLKs) and receptor-like proteins (RLPs) [22,23]. RLKs contain an extracellular ligand recognition domain comprising a leucine-rich repeat sequence (LRR), a transmembrane structural domain (TM), and an intracellular serine/threonine kinase structural domain. In contrast, RLPs are similar to RLKs but lack a cytoplasmic kinase structural domain, rendering them unable to recognize ligand molecules and generate intramolecular or intermolecular phosphorylation [24]. Therefore, RLPs typically function as co-receptors alongside RLKs (such as SOBIR1 and BAK1) to form receptor complexes that cooperatively participate in signaling pathways for immune responses [25,26,27]. Compared to RLKs, fewer studies have been conducted on RLPs. However, an increasing number of findings suggest that RLPs play an important role in plant immunity. The first LRR-RLP identified was the tomato Cf-9 protein, which confers specific resistance to tomato leaf mold fungus carrying the Avr9 effector gene [28,29]. Subsequently, a second LRR-RLP named tomato Cf-4 was shown to be required for resistance to tomato leaf mold fungus expressing Avr4 [30]. In addition, the *Ve1* and *Ve2* genes encoding receptor-like protein-type cell surface receptors in tomatoes are disease-resistant proteins that respond to *Vertucullium wilt* [31,32,33]. Related studies in *Arabidopsis* have also shown that AtRLP30 contributes to resistance to the non-adapted bacterial pathogen *Pseudomonas syringae*, and AtRLP52 is associated with resistance to powdery mildew pathogens [34,35]. The gene *TaRLP1.1* has been identified as a significant defense gene against wheat stripe rust [36]. A previous study in our laboratory also found that the expression level of the receptor-like protein OsRLP1 was affected under RBSDV infection conditions. We revealed that OsRLP1 was able to interact with its junction kinase OsSOBIR1 to mediate the PTI pathway, thus affecting the defense of rice against RBSDV infection [17].

The above research shows that receptor-like proteins play an important role in plant recognition and the perception of external pathogen invasion. However, little is known about the role of receptor-like proteins in plant immune viruses, especially rice–virus interactions. Therefore, further research is needed to investigate the functions and mechanisms of plant pattern recognition receptors in host resistance to viral infection. In this study, we found the receptor-like gene *OsBAP1*, which was markedly induced under SRBSDV infection. We further analyzed the protein structure and subcellular localization of BAP1. Then, viral inoculation of *osbap1-cas* mutants showed that OsBAP1 negatively regulates rice resistance to SRBSDV infection. In addition, we used a high-throughput sequencing approach to gain initial insights into the signaling pathways and functions in which the receptor-like protein OsBAP1 is involved after viral infection. These findings provide new insights into the role of receptor-like proteins in plant immune signaling pathways.

## 2. Materials and Methods

### 2.1. Plant Materials and Growing Conditions

The rice (*Oryza sativa* subsp. *japonica*) varieties used in this study were Wuyujing No.3 and Nipponbare (NIP). We identified and obtained the T3 generation of *osbap1-cas* (CRISPR/Cas9) in a NIP background. All plants were grown in the greenhouse at 28–30 °C with a 14 h/10 h light/dark cycle.

### 2.2. Subcellular Localization Methods

To generate 35S:: OsBAP1-GFP for investigating subcellular localization, the coding sequences of *OsBAP1* were amplified and cloned into pCAMBIA1300 vectors, driven by the CaMV 35S promoter with GFP tag. The subcellular localization assays of 35S:: OsBAP1-GFP were performed using *Agrobacterium strain* GV3101 in *N. benthamiana* cells, and the 35S:: GFP vector was used as a negative control. Briefly, the cultures were centrifuged at 5000× *g* for 2 min to collect the organisms, which were then treated with infiltration buffer (10 mM MgCl_2_, 10 mM MES (pH = 5.6), and 0.2 mM acetosyringone) to a final concentration of OD_600_ = 1.0. The *Agrobacterium* cultures were kept at 28 °C for at least 2 h without shaking. Subsequently, an equal volume of *Agrobacterium* suspension containing 35S:: OsBAP1-GFP or 35S:: GFP was infiltrated into 6-week-old tobacco leaves. After 36 h, the fluorescence signals were captured by confocal laser microscopy (Leica TCS SP10).

### 2.3. Multiple Sequence Alignment and Phylogenetic Analysis

We downloaded the amino acid sequences encoding CDS of OsBAP1 and its highly homologous proteins from rice and *Arabidopsis* for phylogenetic analysis. ClusterlW was used to align all acquired sequences. MEGA6.0 software was used to construct a phylogenetic tree with 1000 bootstrap tests based on the Neighbor Connection (NJ) methods.

### 2.4. Isolation and Identification of Mutant Plants

To generate *osbap1-cas* knockout mutants, the guide RNA sequences of OsBAP1 were selected and cloned into pLYsgRNA-OsU6b to produce rice U6b promoter-driven single-guide RNAs (sgRNAs). The CRISPR/Cas9 plasmids were produced and these constructs were introduced into rice NIP plants by *Agrobacterium tumefaciens*-mediated transformation. T3 homozygous transgenic plants were used for subsequent experiments.

### 2.5. Insect Vector and Virus Inoculation Assays

SRBSDV was transmitted by WBPH (*Sogatella furcifera*). Inoculation of rice with SRBSDV was performed as described previously [37,38]. Previous studies have suggested that WBPHs do not transmit viruses through their eggs. To obtain virus-free WBPHs, adult insects were allowed to feed and lay eggs on Wuyujing No.3 for 3 days. These virus-free WBPH nymphs were used in subsequent experiments. To obtain viruliferous WBPHs, 3 instar nymphs were fed on SRBSDV-infected rice plants for 3–4 days. The nymphs were then transferred to Wuyujing No.3 seedlings and allowed to feed for around 10 days. SRBSDV-infected or virus-free WBPHs were transferred to 10-day-old (3- to 4-leaf stage) wild-type and mutant transgenic rice seedlings (inoculated at a ratio of rice seedlings/insects = 1:3) and allowed to feed for 3–5 days. The insects were removed completely. Plants inoculated with SRBSDV were grown in a greenhouse to observe symptoms. After 30 dpi, disease symptoms and virus accumulation were observed and tested in wild-type and mutant transgenic rice plants. Western blotting and quantitative real-time PCR (RT-qPCR) assays were used to detect the viral accumulation of the plants. Samples of mock and SRBSDV-infected leaves were collected at 30 dpi, and stored at −80 °C for use.

### 2.6. Total RNA Extraction and RT-qPCR Assays

The total RNA of mock and SRBSDV-infected plants was extracted with TRIzol reagent (Invitrogen, Carlsbad, CA, USA) according to the manufacturer’s protocols. Subsequently, total RNA (1–2 µg) was synthesized into cDNA using the fast quant RT Kit (Tiangen, Beijing, China). The RT-qPCR assays were performed using ChamQ SYBR qPCR Master Mix (Without ROX) by the ABI7900HT Sequence Detection System (Applied Biosystems, Carlsbad, CA, USA). The rice actin gene *OsUBQ5* (AK061988) was used to normalize statistics, and the results were analyzed by the 2^−ΔΔCt^ method [39]. The experiment in this study was repeated at least three times and similar results were obtained. The RT-qPCR primer sequences used in this study are shown in Table S1.

### 2.7. Protein Extraction and Western Blotting Analysis

For protein extraction, rice plants infected with SRBSDV were frozen in liquid nitrogen and extracted with lysis buffer (10% SDS, 100 mM Tris-HCl, pH = 6.8). The protein samples were boiled at 100℃ for 10 min in 5× loading buffer (10% SDS, 250 mM Tris-HCl, pH = 6.8, 0.5% BPB, 2% β-Mercaptoethanol, 50% glycerol) and loaded in SDS-PAGE gels for Western blotting assays [40]. Anti-SP10 polyclonal antibody at 1:3000 dilution was used for diagnosis of SRBSDV infection. Total proteins were stained with Ponceau stain to confirm equal loading. The experiments in this study were repeated at least three times and similar results were obtained.

### 2.8. RNA Library Construction and Sequencing

The methods of RNA library construction were described in our previous reports [17,41]. The mock and SRBSDV-infected rice plants were collected at 30 dpi, ground into fine powder, and total RNA extracted using the method described above. Three to five leaves were collected from different seedlings as one biological replicate, and three biological replicates were used for each treatment. The quantity and purity of RNA were assessed and the addition of adapters, size selection, and RNA-seq were all performed by Hangzhou Lianchuan (Hangzhou, China). RNA sequencing utilized the illumina HiSeqTM 2000 platform. Mapping of sequencing reads to the rice genome (The MSU Rice Genome Annotation Project Data base version 7.0) was performed by Bowtie software. Blast2go program was used for data analysis including the gene ontology (GO) functional classes and the Kyoto Encyclopedia of Genes and Genomes (KEGG) pathway. Significant differential expression of genes was assessed using the absolute value of log2 (fold change) ratio ≥ 1 and *p* ≤ 0.05.

### 2.9. Statistical Analysis

Statistical significance were analyzed using one-way ANOVA with Fisher’s least significant difference test. Each experiment was repeated at least three times, and data were represented as means. A *p*-value ≤ 0.05 was considered statistically significant. * at the top of columns indicates significant differences. All analyses were performed using ORIGIN 8.0 software.

## 3. Results

### 3.1. A Gene Encoding a Receptor-like Protein OsBAP1 Was Up-Regulated in Response to SRBSDV Infection

**Figure 1:**
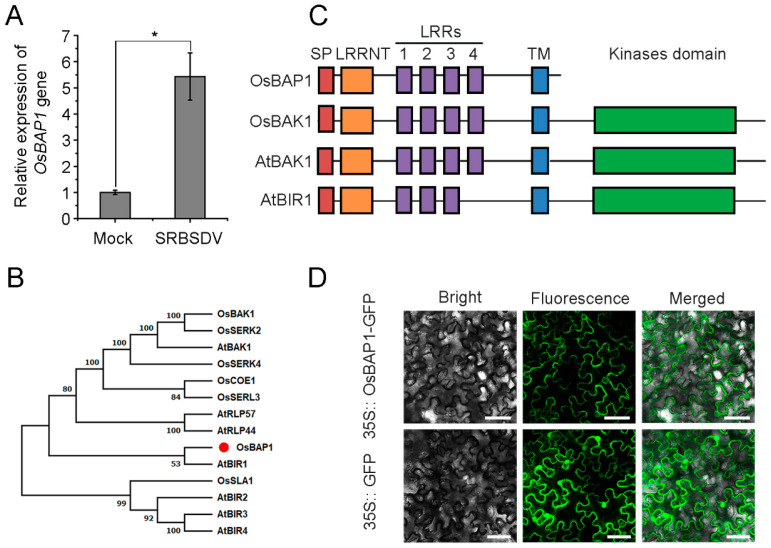
The expression pattern of *OsBAP1* gene: (**A**) RT-qPCR analysis of *OsBAP1* gene expression levels in SRBSDV-infected and control plants at 30 dpi. Values are the means ± SD from 3 biologically independent samples. * at the top of columns indicates significant differences (*p*-value ≤ 0.05) based on Fisher’s least significant difference tests. (**B**) Evolutionary tree analysis of OsBAP1 proteins in rice and *Arabidopsis*. (**C**) Domain prediction for OsBAP1, OsBAK1, AtBAK1, and AtBIR1. The red box represents the signal peptide (SP), the orange box represents leucine-rich repeat N-terminal domains (LRRNT), purple boxes represent leucine-rich repeat domains (LRR), the blue box represents the transmembrane domain (TM), and the green box represents kinase domain (KD). (**D**) Subcellular localization of OsBAP1 protein. 35S:: OsBAP1-GFP was transiently expressed in *N. benthamiana* leaves with 35S:: GFP construct as negative control. Excitation laser wavelengths of 488 nm were used for GFP signals. White bar represents 50 µm.

Previous high-throughput sequencing data revealed that many genes were significantly changed upon viral infection, including the receptor-like protein. Previous studies have demonstrated that RLPs play a pivotal role in plant immunity against viral infection [17]; however, it is not clear whether other receptor-like proteins are involved in this process. Notably, one BAK1-associated receptor gene (called *OsBAP1*) was significantly up-regulated after SRBSDV infection in NIP plants compared to non-infected plants (Figure 1A). A homology analysis of the OsBAP1 protein was conducted using MEGA6.0 software in rice and *Arabidopsis* (Figure 1B). We selected OsBAP1, OsBAK1, and AtBAK1 proteins for domain analysis based on the results of evolutionary tree analysis to further explore the structure and function of OsBAP1. An analysis of the protein sequence showed that OsBAP1 encodes a protein containing a signal peptide, the leucine repeats sequence, and the transmembrane domain while lacking the kinase domain (Figure 1C). To determine the subcellular localization of OsBAP1, we constructed a fusion protein of OsBAP1 with green fluorescent protein (GFP) at the C-terminus, and used 35S:: GFP as a control. The *Agrobacterium*-mediated transient expression system was utilized to introduce the 35S:: OsBAP1-GFP and 35S:: GFP constructs into *N. benthamiana* cells. The subcellular localization results demonstrated that the 35S:: GFP control protein was localized to both the cytoplasm and nucleus, and 35S:: OsBAP1-GFP was found at cytoplasm and plasma membrane (Figure 1D).

### 3.2. OsBAP1 Negatively Regulates Resistance to SRBSDV Infection in Rice

In this study, we investigated the role of OsBAP1 in SRBSDV-infected rice using a reverse genetics approach. To achieve this, we first constructed OsBAP1 knockout mutant transgenic plants using the CRISPR/Cas9 system in the NIP background. DNA sequencing indicated that two independent mutants, *osbap1-cas4* and *osbap1-cas6*, had been generated. The *osbap1-cas4* mutant contained a deletion of GCGT and *osbap1-cas6* with an A insertion, both causing frameshift mutations and a premature stop codon (Figure S1).

**Figure 2:**
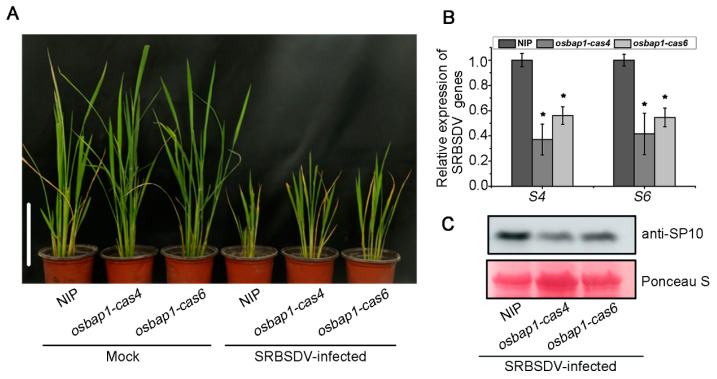
OsBAP1 negatively regulates rice resistance to SRBSDV: (**A**) The symptoms of SRBSDV in WT (NIP) and OsBAP1 mutant (*osbap1-cas4* and *osbap1-cas6*) plants. The phenotypes were photographed at 30 dpi. White bar represents 10 cm. (**B**) The relative expression levels of SRBSDV *S4* and *S6* genes in SRBSDV-infected NIP and *osbap1-cas* plants as detected by RT-qPCR at 30 dpi. UBQ5 was used as the internal reference gene to normalize the relative expression. Values are the means ± SD from 3 biologically independent samples. * at the top of columns indicates significant differences (*p*-value ≤ 0.05) based on Fisher’s least significant difference tests. (**C**) The accumulation of SRBSDV SP10 protein in SRBSDV-infected plants determined by Western blotting at 30 dpi. Total proteins were stained with Ponceau stain to confirm equal loading.

For virus inoculation experiments, two homozygous T3 generation mutant transgenic and control NIP plants were infested with SRBSDV-infected or virus-free insect vector (*Sogatella furcifera*). We then assessed the resistance of mutant transgenic plants to SRBSDV infection by observing disease symptoms and virus accumulation at about 30 dpi. Our inoculation assays showed that SRBSDV-infected plants displayed the symptoms of stunting and significantly increased tillering. The stunting symptoms were more minor in *osbap1-cas* mutants than in NIP controls (Figure 2A). Further analysis of viral accumulation by RT-qPCR assays revealed that the expression levels of SRBSDV *S4* and *S6* genes were significantly lower in *osbap1-cas* mutant plants than in NIP (Figure 2B). In addition, Western blotting analysis showed that the amounts of viral coat protein SP10 were also significantly reduced in *osbap1-cas* mutant transgenic plants compared to wild-type plants (Figure 2C). These results indicated that compared to the NIP control, *osbap1-cas* exhibited enhanced resistance to SRBSDV, suggesting that OsBAP1 plays a negative role in rice resistance to SRBSDV infection.

### 3.3. Differentially Expressed Genes in OsBAP1 Mutant and NIP Plants in Response to SRBSDV Infection

To further investigate the molecular mechanisms of the OsBAP1 protein in SRBSDV-infected rice, we performed high-throughput RNA sequencing (RNA-seq) experiments for the transcriptome analysis of *osbap1-cas6* and NIP plants after SRBSDV infection. Two-week-old rice plants were inoculated with SRBSDV through SRBSDV-infected WBPHs for three days, and a virus-free WBPHs as the negative control. After about 30 days, rice leaves were collected from both mock and SRBSDV-infected plants, and RNA-seq was performed with three biological replicates. Quality control and mapping information is provided in Table S2. Differential expressed gene (DEGs) analysis was performed on the 12 cDNA libraries. The sequencing quality values for Q20% exceeded 99.9%, while Q30% values were from 95% to 99%. The GC content ranged from 47% to 52%. These results show that the sequencing quality was suitable for further analysis.

**Figure 3:**
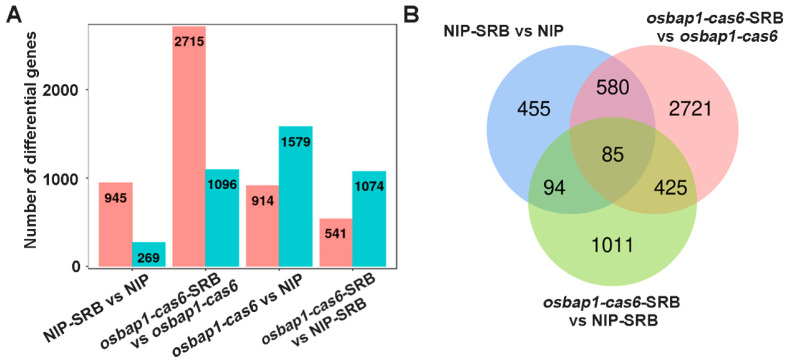
Differentially expressed genes (DEGs) analysis of rice in response to SRBSDV infection: (**A**) Number of DEGs in NIP-SRB vs. NIP (SRBSDV-infected NIP plants compared to NIP control plants); *osbap1-cas6*-SRB vs. *osbap1-cas6* (SRBSDV-infected *osbap1-cas6* plants compared to *osbap1-cas6* plants); *osbap1-cas6* vs. NIP (*osbap1-cas6* plants compared to NIP control plants); and *osbap1-cas6*-SRB vs. NIP-SRB (SRBSDV-infected *osbap1-cas6* plants compared to SRBSDV-infected NIP plants). The pillars in pink represent the numbers of up-regulated genes and down-regulated in blue. (**B**) Venn diagram illustrating the overlapping of DEGs in NIP-SRB vs. NIP, *osbap1-cas6*-SRB vs. *osbap1-cas6*; and *osbap1-cas6*-SRB vs. NIP-SRB.

Based on transcriptome sequencing, we identified many host DEGs at the transcriptional level in SRBSDV-infected rice plants. As shown in Figure 3A,B, a total of 1214 DEGs were identified in NIP plants (NIP-SRB vs. NIP-CK), of which 945 genes were up-regulated and 269 genes were down-regulated. In *osbap1-cas6* plants (*osbap1-cas6*-SRB vs. *osbap1-cas6*-CK), a total of 3811 DEGs were identified, of which 2715 and 1096 genes were significantly up- and down-regulated, respectively. Compared with the NIP-SRB vs. NIP (1214 DEGs), the *osbap1-cas6*-SRB vs. *osbap1-cas6* (3811 DEGs) showed a slight increase. In addition, there were 914 up-regulated genes and 1579 down-regulated genes that differed between *osbap1-cas6* and NIP plants (Figure 3A). A Venn diagram illustrates the number of DEGs specifically affected by SRBSDV infection in the NIP variety (455) and the *osbap1-cas6* (2721) (Figure 3B). The results suggest that the absence of the *OsBAP1* gene in rice induces some differential gene expression after SRBSDV infection. Overall, the results indicate that both viral infection and *OsBAP1* gene knockout cause significant disturbances to the transcriptional levels of a large number of genes in rice.

### 3.4. Analysis of DEGs Induced in OsBAP1 Mutants under SRBSDV Inoculation

**Figure 4:**
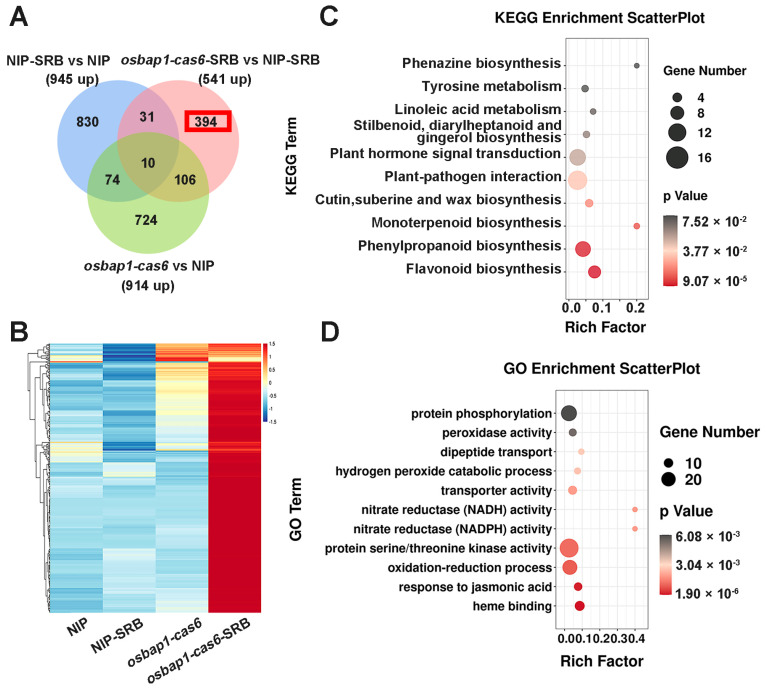
Analysis of the up-regulated genes associated with SRBSDV infection in *osbap1*-*cas* mutant plants: (**A**) Venn diagram illustrating the overlapping of down-regulated 394 DEGs in NIP-SRB vs. NIP, *osbap1*-*cas6*-SRB vs. NIP-SRB, and *osbap1*-*cas6* vs. NIP. (**B**) Hierarchical clustering of 394 DEGs (**A**) based on the log_2_ fold change in transcript levels in NIP and *osbap1*-*cas6* under mock or SRBSDV inoculation. (**C**) KEGG pathway enrichment analysis of 394-up genes (marked with a red rectangle) in (**A**). (**D**) Gene ontology (GO) enrichment analysis of 394-up genes (marked with a red rectangle) in (**A**). “Rich factor” shows the ratio between the number of DEGs and the total genes in this pathway. All differentially expressed genes were selected using the absolute value of log_2_ (fold change) ratio ≥ 1 and *p* ≤ 0.05. Data were collected from three biological replicates, each containing a pool of three plants. SRB, SRBSDV-infected plants.

To further investigate the mechanisms of OsBAP1 negatively regulating rice resistance to SRBSDV, we conducted a comprehensive analysis of the genes that were significantly up-regulated in *OsBAP1* mutants after virus infection. Due to *osbap1-cas* mutants being more resistant to viral infection than NIP plants (Figure 2), we presumed that defense-related genes may be more up-regulated in *osbap1-cas* mutants than that in control plants in response to SRBSDV infection. RNA sequencing analysis showed that defense genes were significantly activated by SRBSDV infection in *osbap1-cas6*, but not in NIP, suggesting that OsBAP1 inhibits the expression of defense genes in rice antiviral immunity. As shown in Figure 4A, the Venn diagram analysis showed 10 overlapping DEGs in all three comparison groups of NIP-SRB vs. NIP-CK, *osbap1-cas6*-SRB vs. *osbap1-cas6*, and *osbap1-cas6*-SRB vs. NIP-SRB. In addition, 541 differential genes were found to be up-regulated in *osbap1-cas6* mutant plants compared to NIP control plants following SRBSDV inoculation, among which 394 differential genes (about 73%) were specifically up-regulated. The heatmap shows that the expression of these genes was effectively higher in the infected mutants than in the other samples (Figure 4B), indicating that these genes might be regulated by OsBAP1 and involved in the rice antiviral defense response.

Therefore, we selected these genes for further biological relationship analysis about their Kyoto Encyclopedia of Genes and Genomes (KEGG) pathways. As shown in Figure 4C, we found that these 394 genes were mainly enriched in various pathways, such as plant-pathogen interactions, plant hormone signal transduction, and phenylpropanoid and flavonoid biosynthesis. It was noteworthy that “plant-pathogen interactions” and “plant hormone signal transduction” were significantly enriched (Figure 4C). Gene Ontology (GO) enrichment analyses revealed that the up-regulated genes were mainly enriched in “protein phosphorylation”, “protein serine/threonine kinase activity”, “oxidation-reduction processes”, and “plant defense responses to jasmonic acid” (Figure 4D). Our previous research has suggested that the jasmonic acid (JA) signaling pathway was involved in antiviral immunity in rice through coordination with other hormonal pathways [40,41,42,43,44,45]. Additionally, receptor-like proteins interact with receptor-like kinases to mediate plant-related immune signals. We believe that OsBAP1 negatively regulates plant immune defense by regulating the expression of defense genes related to these pathways. These results further support the assumption that the deletion of OsBAP1 activates protein phosphorylation, protein kinase activity, and defense-related pathways, especially the JA signaling pathway. Furthermore, our analysis results provide new insight into the gene changes that occur in *osbap1-cas* mutants and NIP plants after SRBSDV infection.

### 3.5. Analysis of DEGs Suppressed in OsBAP1 Mutants under SRBSDV Inoculation

**Figure 5:**
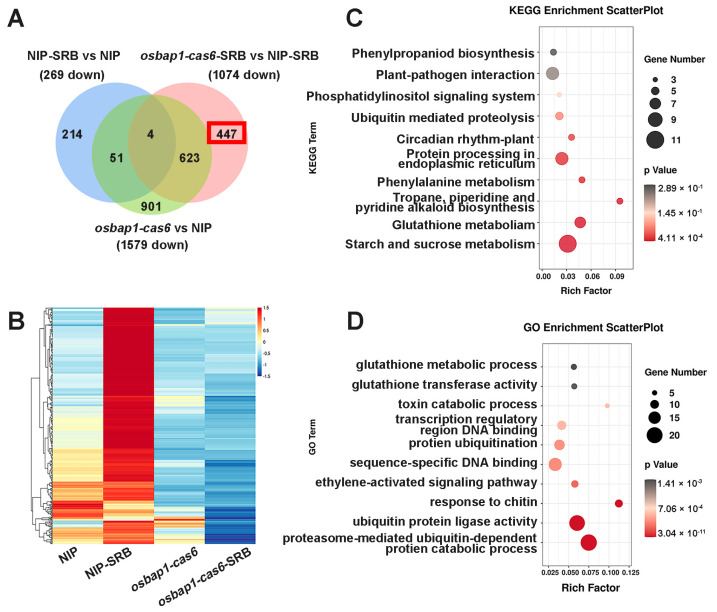
Analysis of the down-regulated genes associated with SRBSDV infection in *osbap1*-*cas6* mutant plants: (**A**) Venn diagram illustrating the overlapping of down-regulated 447 DEGs in NIP-SRB vs. NIP, *osbap1*-*cas6*-SRB vs. NIP-SRB, and *osbap1*-*cas6* vs. NIP. (**B**) Hierarchical clustering of 447 DEGs (A) based on the log_2_ fold change in transcript levels in NIP and *osbap1*-*cas6* under mock or SRBSDV inoculation. (**C**) KEGG pathway enrichment analysis of 447-down genes (marked with a red rectangle) in (**A**). (**D**) Gene ontology (GO) enrichment analysis of 447-down genes (marked with a red rectangle) in (**A**). “Rich factor” shows the ratio between the number of DEGs and the total genes in this pathway. All differentially expressed genes were selected using the absolute value of log_2_ (fold change) ratio ≥ 1 and *p* ≤ 0.05. Data were collected from three biological replicates, each containing a pool of three plants.

In order to better understand the biological function of OsBAP1 in rice–virus interactions, we conducted an analysis of the DEGs that were down-regulated in *osbap1-cas6* mutants and NIP plants after SRBSDV infection. A Venn diagram analysis of the three comparison groups (NIP-SRB vs. NIP-CK, *osbap1-cas6*-SRB vs. *osbap1-cas6*, and NIP-SRB vs. *osbap1-cas6*-SRB) revealed four overlapping DEGs. We observed that 1074 DEGs were down-regulated in *osbap1-cas6* mutant plants compared to NIP plants, with 447 DEGs (approximately 42%) specifically down-regulated (Figure 5A). The heatmap showed that the expression of these genes was significantly lower in the infected mutants than in the other samples (Figure 5B).

We further performed KEGG pathway and GO analyses on these genes. As shown in Figure 5C, the KEGG pathway analysis indicated that these 447 DEGs were mainly concentrated in “starch and sucrose metabolism”, “amino acid metabolism”, and “plant-pathogen interaction” (Figure 5C). Consistent with the above, “plant-pathogen interactions” were significantly enriched. The GO pathway analyses were also used to classify the biological process of the 447 DEGs and showed that these DEGs were mainly enriched in ubiquitin protein ligase activity and metabolic pathways, ethylene activation signaling pathways, and chitin response (Figure 5D). Taken together, these analyses indicate that knockdown of the receptor-like protein OsBAP1 maybe affect the interaction between the plant and SRBSDV by regulating the protein metabolic pathways and the plant hormone signaling pathway in rice.

### 3.6. RT-qPCR Validation of the Expression Profiles of Some Defense Genes

**Figure 6:**
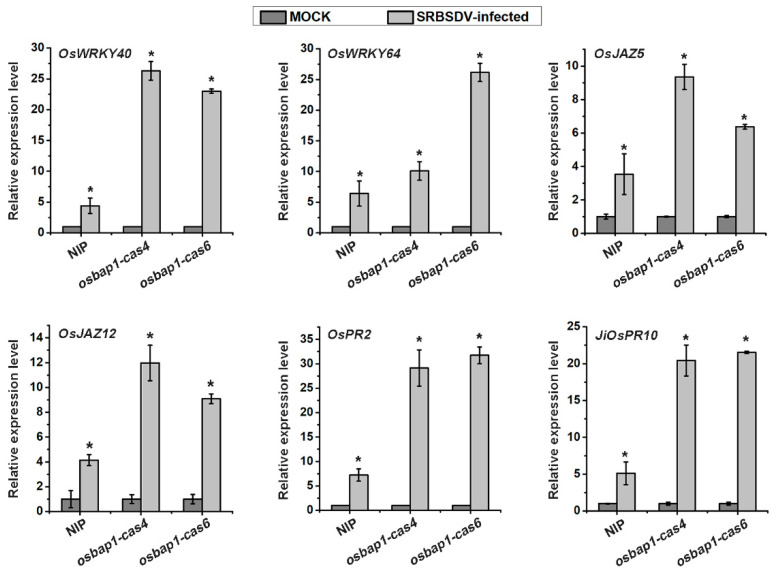
RT-qPCR assays to verify the expression levels of six defense-related genes in SRBSDV-infected NIP and OsBAP1 mutant plants. UBQ5 was used as the internal reference gene to normalize the relative expression. Values are the means ± SD from 3 biologically independent samples. * at the top of columns indicates significant differences (*p*-value ≤ 0.05) based on Fisher’s least significant difference tests.

The transcriptome analysis revealed that certain genes involved in disease resistance pathways, such as the JA signaling pathway and oxidation–reduction response, were up-regulated in *osbap1-cas* plants, but were not activated in NIP plants. It was reported that the transcription factor OsWRKYs could influence the expression of *PR* genes, thereby activating plant defense responses. Our experimental group has conducted extensive studies on the JA signaling pathway and related regulatory factors. JA plays a positive role in the antiviral defense of rice. Therefore, relevant defense genes were selected for RT-qPCR assays, including *OsWRKY40*, *OsWRKY64*, *OsJAZ5*, *OsJAZ12*, *OsPR2*, and *JiOsPR10* genes (Figure 6). To verify the expression patterns of these genes, total RNA was extracted from healthy and SRBSDV-infected *osbap1-cas* mutant and wild-type plants. The results demonstrated that the expression levels of these genes were higher in *osbap1-cas* plants compared to the NIP in response to SRBSDV infection. The RT-qPCR results were consistent with the RNA-seq experiments. These results further suggested that defense-related genes were activated after SRBSDV infection when the receptor-like protein *OsBAP1* was deleted, Further investigation using protein interaction and the construction of various transgenic rice plants is needed to determine how OsBAP1 participates in PTI response and antiviral defense in rice.

## 4. Discussion

Southern rice black-streaked dwarf disease caused by SRBSDV infection has rapidly emerged as a major threat to rice yield in recent years [4,5]. SRBSDV infection causes severe symptoms such as dwarfism and stunted growth in susceptible plants [3]. The recognition of external signals by transmembrane receptors was critical for both plant immunity and development [20]. However, the role of the receptor-like protein in SRBSDV infection remains poorly understood. Therefore, investigating the function of receptor-like proteins in plant immunity and pathogen invasion is very important.

To further elucidate the molecular mechanisms of OsBAP1 protein in rice antiviral defense, this study employed a high-throughput-sequencing approach to conduct an in-depth analysis. The results revealed that there are many DEGs in NIP and *osbap1-cas6* mutant plants with SRBSDV infection (Figure 3). Specifically, genes related to oxidation–reduction response, response to jasmonic acid, and the protein phosphorylation process were significantly induced in *osbap1-cas* mutant plants compared to NIP plants, suggesting that OsBAP1 was mainly involved in regulating the protein metabolic pathways and plant hormone signaling pathway in rice. Many studies have confirmed that the JA pathway plays an essential role in plant antiviral defense [41,42,45,46]. Our previous studies have shown that rice viruses inhibit the JA signaling pathway by suppressing the transcriptional activation activity of the OsMYC transcription factor, thereby facilitating viral infection and attracting insect vectors to facilitate transmission [46]. Moreover, OsGSK2 can directly interact with OsJAZ4 to enhance JA signaling and rice antiviral defense response [43]. These studies further indicate that JA plays a positive role in rice antiviral defense responses. After knocking out OsBAP1 in rice, GO enrichment analyses showed that the up-regulated genes were enriched in “plant defense responses to jasmonic acid” by transcriptome sequencing (Figure 4D). This suggests that OsBAP1 may be involved in a negative regulation of the antiviral network mediated by the JA signaling pathway. Furthermore, the production of reactive oxidative burst is the first step of plant defense response, which serves to transmit signals or directly kill pathogens [47]. This indicates that plant redox has an important role in the disease resistance mechanism. Our results further suggest that OsBAP1 also negatively regulates redox reactions, thereby inhibiting the antiviral immune response of rice (Figure 2 and Figure 3).

Several studies have demonstrated the important role of RLPs in plant–pathogen interactions [29,30]. For instance, AtRLP30 was critical for resisting non-adapted bacterial pathogen *Pseudomonas syringae* in *Arabidopsis* [34,35]. In our laboratory, we have discovered that OsRLP1 interacts with its ligand kinase OsSOBIR1 and positively regulates rice resistance to RBSDV infection [17]. These findings suggest that different types of RLPs have distinct functions in plant defense against pathogen infection. In this study, we identified that the receptor-like protein OsBAP1 plays a negative regulatory role under SRBSDV infection conditions (Figure 2). In addition, the transcriptomic analysis revealed that DEGs related to the protein phosphorylation pathway were enriched in the *osbap1-cas* mutant plants, suggesting that the receptor protein OsBAP1 may interact with its ligand kinase and be involved in the downstream PTI signaling pathway. Previous studies have demonstrated that SOBIR1, a BIR1 inhibitor, specifically interacts with RLPs and was critical for plant growth, development, and defense immunity [48]. It has also been shown that the co-receptor kinase BAK1 interacted with LRR-RLKs and enhanced the downstream signaling pathway [49,50]. Although RLPs can recognize different ligands specifically in the extracellular space, they lacked kinase domains to form signal transduction complexes, which activated signal cascade transmission [17,26,27]. At present, we have only found a regulatory relationship between OsBAP1 and these defense-related genes, playing a negative regulatory role in antiviral immunity. We speculate that OsBAP1 may regulate related immune pathways by interacting with ligand kinases in the PTI pathway. In future work, we aim to screen the ligand kinase that interacts with OsBAP1 protein. Our results show that the receptor protein OsBAP1 may be involved a variety of antiviral defenses in rice and provide a clue for better understanding the role of receptor-like proteins in plant immune signaling pathways.

## Data Availability

All of the materials and data that were used or generated in this study are described and available in the manuscript and supplementary files. The data presented in the study are deposited in the NCBI repository, accession number PRJNA947961.

## Supplementary Materials

The following supporting information can be downloaded at https://www.mdpi.com/article/10.3390/v15040973/s1, Figure S1: Construction of OsBAP1 knockout CRISPR-Cas9 transgenic rice lines. *osbap1-cas4* mutant harbored a deletion of GCGT and *osbap1-cas6* with an insertion of A, which generated a frameshift mutation leading to a premature stop codon. Red asterisk represents premature termination. Table S1: The primers used in RT-qPCR to determine relative expression levels of rice genes. Table S2: Summary of sequencing data quality.
