# Supplementary material for: A Rice Receptor-like Protein Negatively Regulates Rice Resistance to Southern Rice Black-Streaked Dwarf Virus Infection
Source: Viruses. 2023 Apr 15;15(4):973. doi: 10.3390/v15040973 (PMC10141149; doi:10.3390/v15040973)
Supplement: Supplementary file 1 [file viruses-15-00973-s001.zip › viruses-2337070-supplementary/Supplementary/Figure S1.pdf]

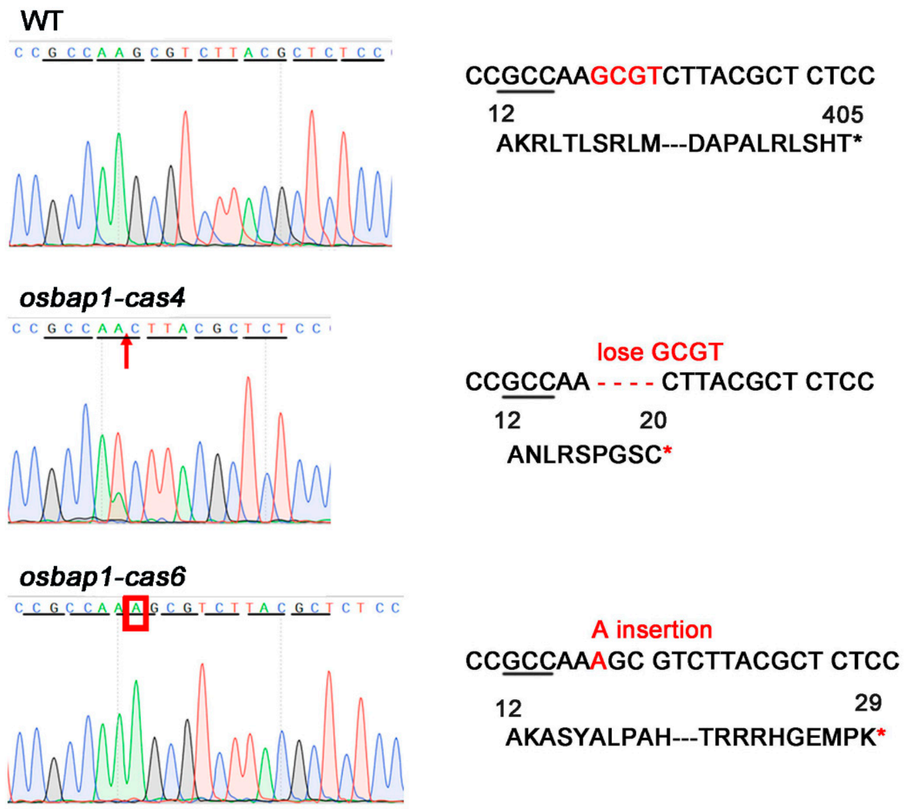

Figure S1. Construction of OsBAP1 knockout CRISPR-Cas9 transgenic rice lines. *osbap1-cas4* mutant harbored a deletion of GCGT and *osbap1-cas6* with an insertion of A, which generating a frameshift mutation leading to a premature stop codon. Red asterisk represents premature termination.
