# Supplementary material for: A Rice Receptor-like Protein Negatively Regulates Rice Resistance to Southern Rice Black-Streaked Dwarf Virus Infection
Source: Viruses. 2023 Apr 15;15(4):973. doi: 10.3390/v15040973 (PMC10141149; doi:10.3390/v15040973)
Supplement: Supplementary file 1 [file viruses-15-00973-s001.zip › viruses-2337070-supplementary/Supplementary/Table S1-S2.pdf]

Table S1. List of primers used for relative expression levels.

Gene	Primer	Primer sequence
OsUBQ5	qRT-UBQ5-F	ACCACTTCGACCGCCACT
	qRT-UBQ5-R	ACGCCTAAGCCTGCTGGTT
OsWRKY40	qRT-OsWRKY40-F	ATCGTGTTGCCGGCGCTTCA
	qRT-OsWRKY40-R	TGGCGAAGAGGCTTGGCGTTGT
OsWRKY64	qRT-OsWRKY64-F	GTGCAGTCAGGTATTGGTCC
	qRT-OsWRKY64-R	TTTGAGCTTCCAGACCCCAT
OsJAZ5	qRT-OsJAZ5-F	CTCCTCTTCCTCCTCCATCC
	qRT-OsJAZ5-R	TCTGCCTTATCGTGGGAGAC
OsJAZ12	qRT-OsJAZ12-F	TCTTCTACGACGGGAGGATG
	qRT-OsJAZ12-R	TCTGTGGTGCCTGATACTGTG
OsPR2	qRT-OsPR2-F	CATCAGCCTCAACTACGCCA
	qRT-OsPR2-R	GCTTCTGGTTGTACGTCTGC
JiOsPR10	qRT-JiOsPR10-F	GACAAGTGCGAGTGCAAGTC
	qRT-JiOsPR10-R	AGGGACTCCTTAGCCTTGGT
SRBSDV S4	qRT-SRBSDV S4-F	AAAGTGAACCCGTTGCTGAC
	qRT-SRBSDV S4-R	TGCAACGCTAGATCCTATGC
SRBSDV S6	qRT-SRBSDV S6-F	ATCTGCTTTTCCCCTTCCGA
	qRT-SRBSDV S6-R	GATTCCGCGTTTGAAGAGTCA
OsBAP1	qRT-OsBAP1-F	GCCTCTCCACGACTCCAATC
	qRT-OsBAP1-R	TCATTTGGGCATCTCACCGT
OsBAP1	Lic-OsBAP1-F	CgACgACAAgACCgTCACCatgTCTTTTG ACACCGGGCT
	Lic-OsBAP1-R	GAggAgAagAgCCgTCgTGTGTGTG ATAGACGTAGGG

Table S2. Summary of sequencing data quality.

Sample	Raw Data	Valid Data	Valid Ratio	Q20%	Q30%	GC content%
*osbap1-cas6*_CK1	41443250	34686930	83.70	99.98	98.26	50
*osbap1-cas6*_CK2	41884338	36429076	86.98	99.97	98.14	50
*osbap1-cas6*_CK3	41155734	34090536	82.83	99.97	98.09	50
*osbap1-cas6*_SRB1	35233198	34400650	97.64	99.97	97.96	48.50
*osbap1-cas6*_SRB2	53719432	50081740	93.23	99.97	97.13	49.50
*osbap1-cas6*_SRB3	53092928	51102278	96.25	99.97	98.28	50.50
NIP_CK1	48289724	41029012	84.96	99.98	98.18	48
NIP_CK2	51191196	48529394	94.80	99.96	96.71	48.50
NIP_CK3	54031174	52803284	97.73	99.98	98.01	48
NIP_SRB1	35660708	34955720	98.02	99.97	97.92	48
NIP_SRB2	36408324	35721242	98.11	99.98	97.93	48.50
NIP_SRB3	41882818	36398420	86.91	99.97	97.92	50
